# Supplementary material for: Mechanisms governing the pioneering and redistribution capabilities of the non-classical pioneer PU.1
Source: Nat Commun. 2020 Jan 21;11:402. doi: 10.1038/s41467-019-13960-2 (PMC6972792; doi:10.1038/s41467-019-13960-2)
Supplement: Supplementary file 7 — Source data [file 41467_2019_13960_MOESM7_ESM.zip › Source_Data/Figure6/Figure6B_MotifScanOutput/FLI1_PU.1/homerResults/motif36.similar.html]

motif36

## Information for motif36

A
T
C
G
C
G
T
A
A
G
T
C
A
C
T
G
A
C
G
T
A
T
G
C
C
G
T
A
A
G
T
C
  
Reverse Opposite:  

C
T
A
G
A
C
G
T
A
T
C
G
C
G
T
A
A
G
T
C
A
C
T
G
A
C
G
T
T
A
G
C
  

|  |  |
| --- | --- |
| p-value: | 1e-27 |
| log p-value: | -6.269e+01 |
| Information Content per bp: | 1.857 |
| Number of Target Sequences with motif | 360.0 |
| Percentage of Target Sequences with motif | 3.62% |
| Number of Background Sequences with motif | 627.4 |
| Percentage of Background Sequences with motif | 1.72% |
| Average Position of motif in Targets | 96.8 +/- 57.3bp |
| Average Position of motif in Background | 92.0 +/- 60.9bp |
| Strand Bias (log2 ratio + to - strand density) | 0.3 |
| Multiplicity (# of sites on avg that occur together) | 1.04 |
| Motif File: | file (matrix) reverse opposite |

### Similar de novo motifs found

|  |  |  |  |  |  |  |  |
| --- | --- | --- | --- | --- | --- | --- | --- |
| Rank | Match Score | Redundant Motif | P-value | log P-value | % of Targets | % of Background | Motif file |
| 1 | 0.822 | C T A G A G C T A C T G G T C A A T G C A C T G A C G T G T A C C G T A | 1e-21 | -49.090944 | 1.58% | 0.55% | motif file (matrix) |
| 2 | 0.802 | G A C T C A T G T G C A G T A C C T A G G C A T A G T C C G T A G A C T T G A C | 1e-21 | -48.806795 | 1.51% | 0.50% | motif file (matrix) |
| 3 | 0.781 | T C G A A C G T A C T G C G T A G A T C C T A G G C A T G T A C C G T A A G C T G T A C | 1e-17 | -40.234467 | 1.42% | 0.52% | motif file (matrix) |
